# Sensory migraine aura is not associated with structural grey matter abnormalities

**DOI:** 10.1016/j.nicl.2016.02.007

**Published:** 2016-02-17

**Authors:** Anders Hougaard, Faisal Mohammad Amin, Nanna Arngrim, Maria Vlachou, Vibeke Andrée Larsen, Henrik B.W. Larsson, Messoud Ashina

**Affiliations:** a Danish Headache Center and Department of Neurology, Rigshospitalet Glostrup, Faculty of Health and Medical Sciences, University of Copenhagen, Denmark; b Department of Radiology, Rigshospitalet Blegdamsvej, Denmark; c Functional Imaging Unit, Department of Clinical Physiology, Nuclear Medicine and PET, Rigshospitalet Glostrup, Faculty of Health and Medical Sciences, University of Copenhagen, Denmark

## Abstract

Migraine with aura (MA) is characterized by cortical dysfunction. Frequent aura attacks may alter cerebral cortical structure in patients, or structural grey matter abnormalities may predispose MA patients to aura attacks. In the present study we aimed to investigate cerebral grey matter structure in a large group of MA patients with and without sensory aura (i.e. gradually developing, transient unilateral sensory disturbances). We included 60 patients suffering from migraine with typical visual aura and 60 individually age and sex-matched controls. Twenty-nine of the patients additionally experienced sensory aura regularly. We analysed high-resolution structural MR images using two complimentary approaches and compared patients with and without sensory aura. Patients were also compared to controls. We found no differences of grey matter density or cortical thickness between patients with and without sensory aura and no differences for the cortical visual areas between patients and controls. The somatosensory cortex was thinner in patients (1.92 mm vs. 1.96 mm, P = 0.043) and the anterior cingulate cortex of patients had a decreased grey matter density (P = 0.039) compared to controls. These differences were not correlated to the clinical characteristics. Our results suggest that sensory migraine aura is not associated with altered grey matter structure and that patients with visual aura have normal cortical structure of areas involved in visual processing. The observed decreased grey matter volume of the cingulate gyrus in patients compared to controls have previously been reported in migraine with and without aura, but also in a wide range of other neurologic and psychiatric disorders. Most likely, this finding reflects general bias between patients and healthy controls.

## Highlights

• Migraine aura per se is not associated with altered grey matter structure.
• Migraine patients have decreased cingulate cortical grey matter.
• This finding likely reflects general bias between patients and healthy controls.

## 1 Introduction

The hallmark of migraine with aura (MA) is recurrent attacks of transient cortical dysfunction (Headache Classification Committee of the International Headache Society (IHS), 2013). The most common migraine aura symptoms are visual and sensory disturbances (Russell and Olesen, 1996). The pathological features that specifically predispose MA patients to aura attacks, and the effects on the brain from repeated MA attacks are largely unknown. Recently, magnetic resonance imaging (MRI) studies have suggested a relation between aura and altered grey matter structure (Granziera et al., 2013, Granziera et al., 2006). MA patients definitively have *dysfunctional* cortex during, (Hadjikhani et al., 2001) and likely also between, (Datta et al., 2013, Hougaard et al., 2014b) attacks. Therefore, MA patients could indeed be expected to have specific structural grey matter abnormalities accompanying abnormal neuronal functioning. Such abnormalities could hypothetically represent cortex that is more susceptible to eliciting waves of cortical spreading depression (CSD), the underlying pathophysiological phenomenon of the aura (Granziera et al., 2006), or they could be a result of frequent CSD episodes. In animals, CSD has been shown to alter cortical microstructure (Dreier, 2011).

We previously investigated interhemispheric structural grey matter differences in a selected group of MA patients with frequent visual aura consistently occurring in the same hemifield and found no structural changes in the contralateral hemisphere (Hougaard et al., 2015a). However, MA patients may exhibit structural brain abnormalities on the whole-brain level or changes may be present only in subgroups of patients, depending on the aura symptoms.

In the present study, we investigated a large group of MA patients with visual aura in the interictal phase. Approximately half of these patients, in addition, experienced sensory aura regularly. We further included a group of carefully individually matched healthy controls. State of the art high-field T1 weighted MRI was applied to investigate cortical and subcortical grey matter structure in these groups. We hypothesised that MA patients with visual and sensory aura exhibit structural differences compared to patients with visual aura only, and that grey matter structure of MA patients is different from that of matched controls.

## 2 Methods

### 2.1 Study design

We used two complementary approaches to investigate differences in grey matter structure between MA patient subgroups and in patients vs. controls:

1) Voxel-based morphometry (VBM) and 2) Surface-based morphometry (SBM). VBM gives a mixed measure of grey matter volume and density, while SBM can selectively estimate cortical thickness (Hutton et al., 2009). Thus, a combination of these two methods is useful to both detect and specify the underlying grey matter changes. For both methods we applied whole-brain analyses as well as analyses of pre-selected regions of interest (ROIs). All analyses were carried out with and without the addition of nuisance variables: age, gender, attack frequency and disease duration.

### 2.2 Patients and controls

We recruited 60 patients (42 F, 18 M, mean age 33.36 years [range 18–59 years]) suffering from migraine with typical aura (MA) according to the second edition of The International Classification of Headache Disorders (The International Classification Of Headache Disorders, 2nd edition, 2004). We also included 60 individually age and sex matched healthy controls (42 F, 18 M, mean age 33.39 years [range 18–59 years]). Data from 20 of these patients have been included in three previously published studies (Hougaard et al., 2014b, Hougaard et al., 2015a, Hougaard et al., 2015b), data from 20 of the controls have been included in two previous studies (Hougaard et al., 2014b, Hougaard et al., 2015b), while data from another 20 patients and 20 controls have been included in one study (Hougaard et al., 2015b). The remaining patients (n = 20) and controls (n = 20) were included for the present study specifically. Exclusion criteria for both groups were any other type of headache except infrequent tension-type headache, serious somatic or psychiatric conditions, or intake of daily medication including prophylactic migraine treatment. We excluded controls if they had a history of any type of migraine or first-degree relatives with a history of any type of migraine (including, but not limited to migraine with aura). A thorough medical history was taken in patients and controls and all participants underwent a complete neurological examination.

The median MA attack frequency was 12 attacks per year [range 2–96 attacks/year]. Prior to inclusion, patients gave a very detailed description of their aura symptoms. All patients reported visual aura symptoms in every attack. Twenty-nine patients additionally experienced typical sensory aura regularly (in one third of attacks or more) (7 males, median MA attack frequency 36 attacks/year, mean age 32.22 years) while the remaining 31 patients reported visual aura only (11 males, median MA attack frequency 12 attacks/year, mean age 34.43 years). All patients experienced headache following aura episodes. All patients were migraine free at least 48 h before and after the MRI scan.

The Ethics Committee of the County of Copenhagen (H-KA-20060083) approved the study, which was undertaken in accordance with the Helsinki Declaration of 1964, as revised in 2008. The study was carried out at Glostrup Hospital, Copenhagen Area, Denmark between April 2011 and June 2014. All subjects gave written informed consent to participate in the study.

## 3 MRI procedure

MRI was performed on a 3.0 T Philips Intera Achieva scanner (Philips Medical Systems, Best, The Netherlands) using a 32-element phased-array receive head coil. Anatomical images were acquired using a T1-weighted three-dimensional turbo field-echo sequence (170 sagittal slices of 1 mm thickness; in-plane resolution 1 × 1 mm; repetition time 9.9 s; echo time 4.6 ms; and flip angle 81 degrees). The T1-weighted images were reviewed by an experienced neuroradiologist (VAL), who did not find structural abnormalities in any of the subjects.

## 4 Data analysis

### 4.1 Voxel-based morphometry

Structural data were analysed with FSL-VBM (http://fsl.fmrib.ox.ac.uk/fsl/fslwiki/FSLVBM) (Douaud et al., 2007), an optimized VBM protocol (Good et al., 2001) carried out with FSL tools (Smith et al., 2004). First, structural images were brain-extracted and grey matter segmentation was performed before the images were registered to the Montreal Neurological Institute (MNI) 152 standard space using non-linear registration. The resulting images were averaged to create a left–right symmetric, study-specific grey matter template. Second, all native grey matter images were non-linearly registered to this study-specific average template and “modulated” to correct for local expansion or contraction due to the non-linear component of the spatial transformation. The modulated grey matter images were then smoothed with an isotropic Gaussian kernel with a sigma of 3 mm, i.e. approximately full width half maximum (FWHM) of 3 × 2.3 = 6.9 mm. We compared 1) patients with vs. patients without sensory and 2) patients vs. matched controls aura in a two-group design in a voxel-wise general linear model (GLM) using permutation-based non-parametric testing (Nichols and Holmes, 2002), correcting for multiple comparisons across space by threshold-free cluster enhancement (Smith and Nichols, 2009) (cluster-wise P < 0.05). Age, gender, disease duration, and attack frequency were included in the model as nuisance variables.

### 4.2 Surface-based morphometry

Cortical reconstruction and volumetric segmentation was performed with the FreeSurfer image analysis suite (http://surfer.nmr.mgh.harvard.edu/) (Dale et al., 1999, Fischl and Dale, 2000). Using this approach, the grey and white matter surfaces were defined by an automated brain segmentation process. An experienced investigator, who was blinded with respect to whether subjects were patients or controls, then manually corrected the automated segmentation. Cortical thickness was estimated at each point across the cortex by calculating the distance between the grey/white matter boundary and the cortical surface. Individual whole brain surface maps were then registered to a common FreeSurfer template surface (fsaverage) by the FreeSurfer spherical registration system (Fischl et al., 1999) and smoothed with a 10 mm 2D Gaussian smoothing kernel (Fischl et al., 1999). We compared a) patients with vs. patients without sensory aura and b) patients vs. controls in a vertex-wise GLM using an unpaired design while applying cluster-wise correction for multiple comparisons using a permutation-based non-parametric analysis (cluster-wise P < 0.05). Age, gender, disease duration, and attack frequency were included in the model as nuisance variables. Additionally, we measured the volume of the thalamus, caudate, putamen, amygdala and hippocampus bilaterally for each subject, as well as the total intracranial volume, total grey matter volume, total cortical volume, and total subcortical grey matter volume using FreeSurfer's automated segmentation.

### 4.3 Region of interest analysis

Several regions of interest (ROIs) were examined for focal changes of grey matter using both of the methods described above. We investigated the following ROIs parcellated by FreeSurfer: Primary visual cortex (V1), secondary visual cortex (V2), visual area V5/MT and somatosensory cortex (SSC, Brodmann areas BA1, BA2, BA3a and BA3b). Furthermore, we examined the following ROIs, which are not available in FreeSurfer, using the FSL versions of the Harvard-Oxford atlases and the Jülich histological atlas (Eickhoff et al., 2006): Visual area V3, visual area V4 and the lateral geniculate nucleus (LGN) of the thalamus, whole thalamus, insula, inferior frontal gyrus (IFG), anterior cingulate cortex, and posterior cingulate cortex. We investigated these specific ROIs since visual aura may be related to alterations of visual areas (V1–V5, LGN) while sensory aura may be related to areas of sensory function (SSC, thalamus). Previous VBM studies have reported migraine-related grey matter changes in insula, IFG, and cingulate cortex (Hougaard et al., 2014a, Hougaard et al., 2015a). Statistical analyses were carried out as described for VBM above, while restricting the voxel-wise calculations to voxels within each ROI. Age, gender, disease duration, and attack frequency were included in the model as nuisance variables.

### 4.4 Additional analyses

Cortical density and cortical thickness has previously been shown to decrease linearly with age (Good et al., 2001, Salat et al., 2004). In order to assess the sensitivity of the methods used in this study, we investigated the relation of mean whole-brain density and total cortical grey matter volume with subject age. Statistical testing for correlation was applied using Pearson's product–moment correlation.

We also assessed if the age-related grey matter changes differed between patients and controls by testing for differences in the age-grey matter interaction between the groups.

## 5 Results

We found no differences between MA patients with (N = 29) and without (N = 31) sensory aura for any of the applied analyses. Thus, the presence of sensory aura was not associated with grey matter differences in SBM or VBM analyses on the whole-brain level or for any of the investigated cortical or subcortical ROIs, including the somatosensory cortex.

The whole-brain VBM and SBM analyses showed no differences between patients and controls. In the whole-brain analyses of the patients specifically, we found no areas where cortical thickness or density were dependent on attack frequency or disease duration, when correcting for the effects of age and gender.

**Fig. 1:**
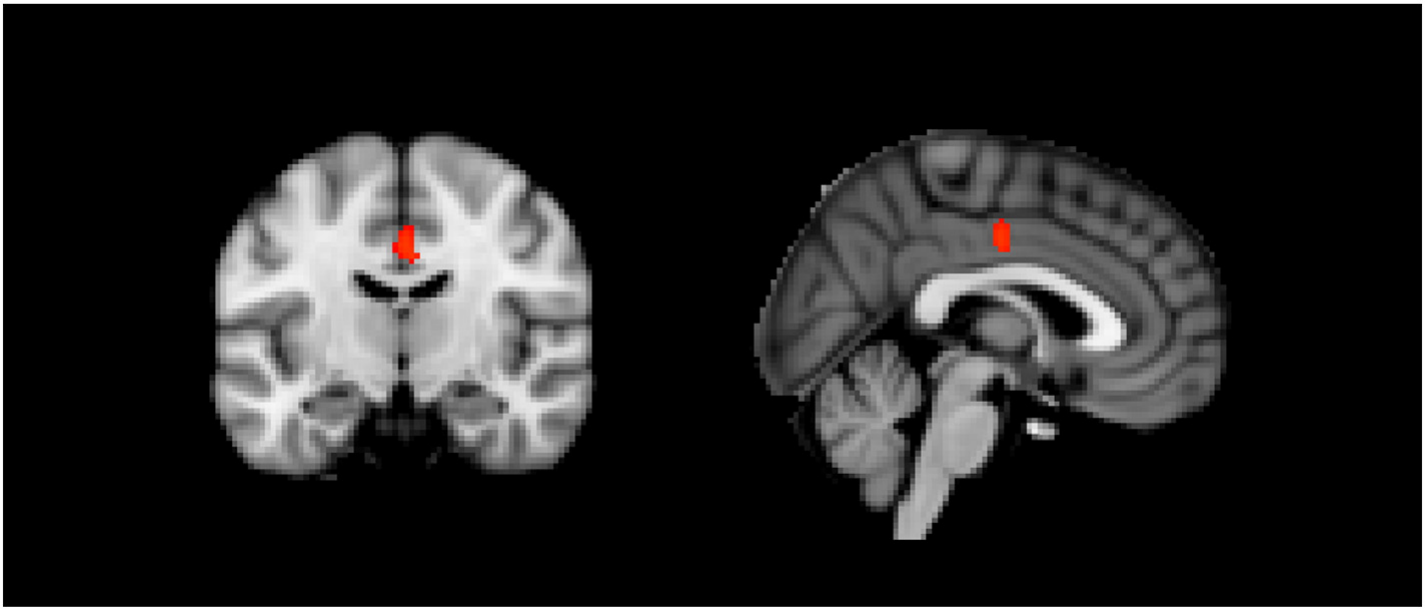
Area of reduced grey matter in the cingulate cortex of patients (N = 60) versus matched controls (N = 60).

Using the ROI-based approach, we found no differences for any of the visual cortical areas between patients with visual aura (N = 60) and matched controls. Significantly decreased VBM grey matter values were found bilaterally in the dorsal part of the anterior cingulate cortex of patients compared to controls (P = 0.039, MNI coordinates (x,y,z,) = (− 2, − 12, 38)). See Fig. 1. When corrected for the effects of age and gender, the grey matter density of this area was not related to attack frequency or disease duration.

**Fig. 2:**
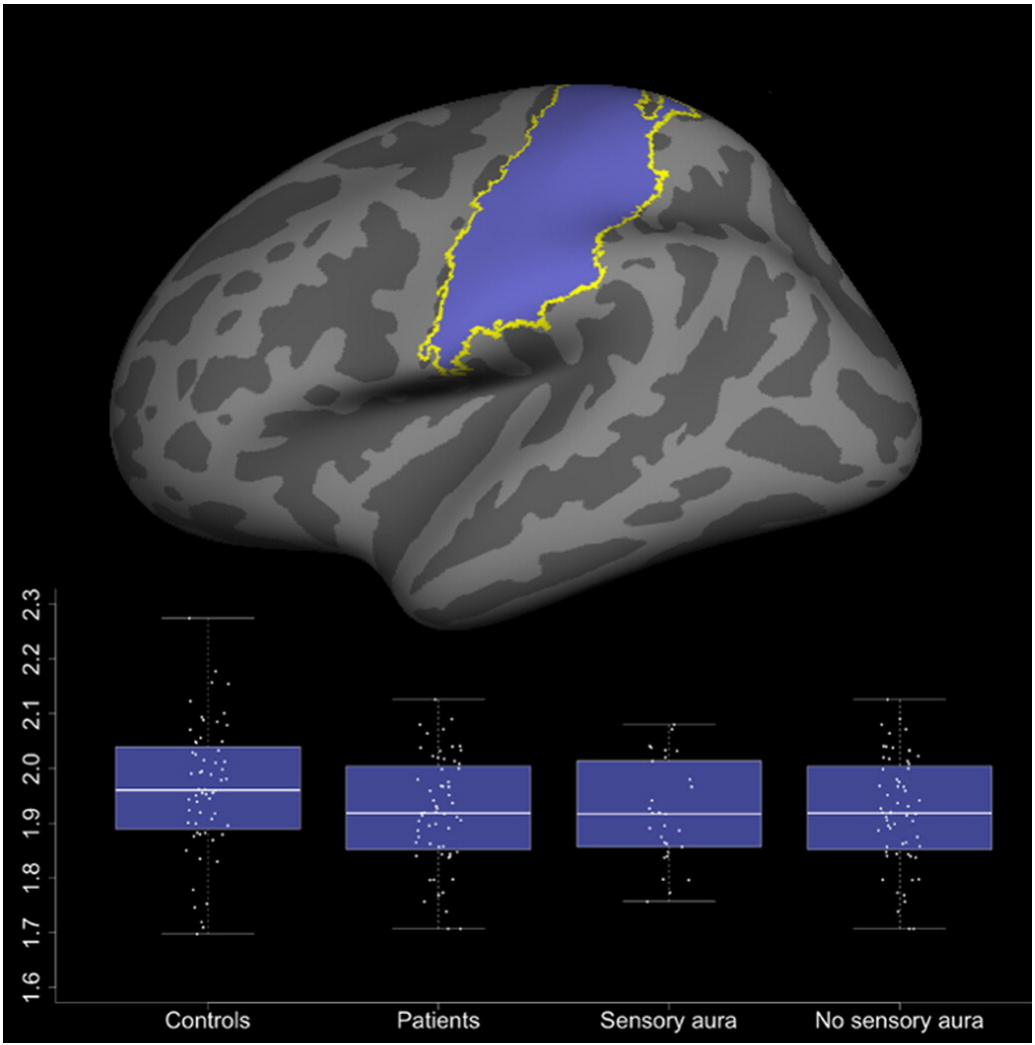
Cortical thickness of the somatosensory cortex in controls, all patients and in patient subgroups. The top image shows the investigated region of interest.

The somatosensory cortex (SSC) was thinner in patients compared to controls (1.92 mm vs. 1.96 mm, P = 0.043, see Fig. 2). When corrected for age and gender effects, the cortical thickness of this area was not related to attack frequency or disease duration. To control for differences in cranial size, we added the total intracranial volume, as obtained from the automated segmentation, to the model. There was a significant effect of the intracranial volume (P = 0.008), but this only slightly affected the between-group difference (P = 0.047). One healthy subject had an extreme SSC thickness (2.27 mm; mean of all subjects: 1.94 mm, SD: 0.11). The between-group difference was not significant when leaving this subject out (P = 0.10).

We found no overall difference in intracranial volume between patients and controls (1088 cm^3^ vs. 1107 cm^3^, P = 0.51). We specifically compared cortical density (VBM) and thickness (SBM) between patient subgroups with and without sensory aura for the SSC and other ROIs as specified above. These analyses showed no differences for any ROIs.

As an exploratory analysis, we measured the cortical thickness specifically in the cingulate cortex area that was abnormal in the VBM analysis, and found a thinner cortex in patients compared to controls in this area specifically (2.75 mm vs. 2.86 mm, P = 0.02).

Volumes of subcortical structures did not differ between subject groups. We found no difference in total grey matter volume for comparison of patients vs. controls (640 cm^3^ vs. 650 cm^3^, P = 0.4), cortical volume (464 cm^3^ vs. 474 cm^3^, P = 0.3), or subcortical grey matter volume (176 cm^3^ vs. 177 cm^3^, P = 0.78). We found highly significant correlations between age and whole-brain grey matter density (VBM, P = 2.6 ∗ 10^− 16) and whole-brain cortical volume (SBM, P = 0.8 ∗ 10^− 9). The relation between age and grey matter structure did not differ between patients and controls in the VBM or SBM analyses.

## 6 Discussion

We conducted the largest study to date specifically investigating grey matter structure in migraine with aura. Most importantly, we found no abnormalities of cortical or subcortical grey matter structure when comparing migraine patients with and without sensory aura. Although it is clinically evident that the somatosensory cortex is dysfunctional during aura episodes in patients with sensory aura, we found no differences between these subgroups when specifically investigating this cortical area. Likewise, we found no differences in the cortical visual areas between the whole group of sixty patients, all experiencing regular visual auras, compared to controls. This, in our interpretation, strongly indicates that cerebral cortex specifically susceptible to CSD, and subjected to regular episodes of CSD, is *not* structurally different from cortex without these features. Thus, migraine aura per se is unlikely to be associated with macro- or mesostructural grey matter abnormalities. These results are in line with our previous study in patients with side-locked visual aura showing no structural abnormalities of grey matter in the hemispheres contralateral to the perceived aura (Hougaard et al., 2015a).

Our results from comparison of patients to controls confirm previous findings of decreased cingulate cortical grey matter, which is the most consistent finding in VBM studies of *mixed* populations of migraine patients with and without aura (Hougaard et al., 2014a). Reduced grey matter of the cingulate cortex is *not* specific for migraine. This abnormality has been reported previously in very different disorders: chronic pain conditions, substance abuse, and a wide range of neurological and psychiatric disorders (see Table 1). Puzzlingly, since nearly all studies in Table 1 compared patients to healthy controls, reduced VBM values of the cingulate cortex appears to be an indicator of disease in general. Currently, it is not fully understood what VBM changes reflect exactly. Histological measures, such as neuronal density, do not correlate well with VBM grey matter probability maps (Eriksson et al., 2009) and changes in cerebral blood flow produce “apparent” grey matter volume changes in VBM analyses (Franklin et al., 2012). We further demonstrated cortical thinning of this area using the complementary method of SBM, thus supporting that decreased VBM values in this case indeed reflect grey matter reduction. In the present study we not only compared patients to controls, but we also compared two subgroups of MA patients, with and without sensory aura, thus likely avoiding this potential confounder of “general health status”.

In the current analysis, we further found decreased cortical thickness of the SSC in patients compared to controls. However, this difference was extremely small (0.04 mm) and largely driven by a small between-group difference in intracranial volume and by one outlier in the healthy control group. While it cannot be ruled out that this small effect is somehow related to migraine pathophysiology, e.g. through adaptation of cortical areas involved in pain-processing, we were unable to demonstrate a correlation of this cortical thinning to migraine attack frequency or time since diagnosis, suggesting that this is not the case. Previous SBM reports of SSC thickness in migraine have yielded highly controversial results (see Table 2). Collectively, there is no firm evidence at present of altered SSC thickness in migraine with or without aura. Cortical thickness is highly variable and likely dependent on many factors, e.g. age and gender (Luders et al., 2006, Salat et al., 2004). Such confounders must be taken into account in order to improve future SBM and VBM studies in migraine as well as other conditions.

In conclusion, migraine aura symptoms per se are not associated with altered cortical or subcortical grey matter. Furthermore, this study supports the existing evidence that alterations of grey matter structure can be demonstrated in migraine patients, most consistently a grey matter reduction of the cingulate cortex, when compared to healthy controls. However, similar abnormalities have been reported in a wide range of disorders (Table 1), suggesting that these are not migraine-specific.

## Potential conflicts of interest

Dr. Hougaard, Dr. Amin, Dr. Arngrim, Dr. Vlachou, Dr. Larsen, and Dr. Larsson report no disclosures. Dr. Ashina is a consultant or scientific adviser for Allergan, Amgen, Alder, ATI, and Eli Lilly.

## Figures and Tables

**Table 1:** Voxel-based morphometry studies reporting grey matter reduction of the cingulate gyrus in various disorders. ACC: Anterior cingulate cortex. MCC: Middle cingulate cortex. PCC: Posterior cingulate cortex.

Study	Disorder	Location
Franklin et al., Biol Psychiatry 2002	Cocaine abuse	Right ACC
Job et al., NeuroImage 2002	Schizophrenia	Right ACC
Yamasue et al., PNAS 2003	Posttraumatic stress disorder	Right ACC
Wilke et al., Psychiatry Res 2004	Bipolar disorder	Left ACC
Schmidt-Wilcke et al., Neurology 2005	Chronic tension type headache	Bilateral ACC and right PCC
Valente Jr. et al., Biol Psychiatry 2005	Obsessive–compulsive disorder	Left ACC
Pennanen et al., J Neurol Neurosurg Psychiatry 2005	Mild cognitive impairment	Left ACC
Prinster et al., NeuroImage 2006	Multiple sclerosis	Bilateral ACC
Draganski et al., NeuroImage 2006	Phantom pain	Right PCC and left ACC
Mueller et al., Epilepsia 2006	Temporal lobe epilepsy	Ipsilateral PCC
Mühlau et al., Am J Psychiatry 2007	Anorexia nervosa	Bilateral ACC
Tang et al., Psychiatry Res 2007	Major depressive disorder	Bilateral ACC
Kuchinad et al., J Neurosci 2007	Fibromyalgia	
Uchida et al., Psychiatry Res 2008	Panic disorder	Left MCC
Rodriguez-Raecke et al., J Neurosci 2009	Hip osteoarthritis	Bilateral ACC
Liu et al., Psychiatry Clin Neurosci 2009	Heroin dependence	Bilateral ACC
Müller-Vahl et al., BMC Neuroscience 2009	Tourette syndrome	Bilateral ACC
Zhou et al., Eur J Radiol 2011	Internet addiction	Left ACC and left PCC
Amico et al., Psychiatry Res 2011	Attention deficit hyperactivity disorder	Bilateral ACC
Husain et al., Brain Res 2011	Hearing loss	Right ACC
Mordasini et al., J Urol 2012	Chronic pelvic pain syndrome	Left ACC
Agostini et al., Neurogastroenterol Motil 2013	Crohn's disease	Left ACC, right MCC, left PCC
Nenadic et al., Psychiatry Res 2015	Narcissistic personality disorder	Bilateral ACC
Present study	Migraine with typical aura	Bilateral ACC

**Table 2:** Surface-based morphometry studies of somatosensory cortical thickness in migraine patients.

Study	Subjects	Reported findings
DaSilva et al., Neurology 2007	MA: 12, MO: 12, HC: 12	Thicker SSC in migraine patients (MA and MO) vs. HC
Datta et al., Cephalalgia 2011	MA: 28:, MO: 28, HC: 28	No differences between groups
Maleki et al., Brain 2012	(MA + MO): 11, HC: 11	No difference in SSC thickness between groups
Maleki et al., Cephalalgia 2012	LF = (1 MA + 9 MO with attack frequency 1–2/month), HF = (1 MA + 9 MO with attack frequency 8–14/month), HC: 20	SSC thickness: HF > LF, HC > LF, HF > HC
Messina et al., Radiology 2013	MA: 32:, MO: 31, HC: 18	No differences in SSC thickness between groups
Magon et al., J Headache Pain 2014 (abstract)	MA: 39, MO: 92, HC: 115	Thinner SSC in migraine patients (MA and MO) vs. HC
Kim et al., Cephalalgia 2014	MO: 56, HC: 34	Thicker SSC in migraine patients vs. HC
Present study	MA: 60, HC: 60	Thinner SSC in migraine patients vs. HC

MA: Migraine with aura, MO: Migraine without aura, HC: Healthy controls, LF: Low frequency, HF: High frequency, SSC: Somatosensory cortex.
